# Electronic Structure of Kramers Nodal‐Line Semimetal YAuGe and Anomalous Hall Effect Induced by Magnetic Rare‐Earth Substitution

**DOI:** 10.1002/advs.202501669

**Published:** 2025-05-08

**Authors:** Takashi Kurumaji, Jorge I. Facio, Natsuki Mitsuishi, Shusaku Imajo, Masaki Gen, Motoi Kimata, Linda Ye, David Graf, Masato Sakano, Miho Kitamura, Kohei Yamagami, Kyoko Ishizaka, Koichi Kindo, Taka‐hisa Arima

**Affiliations:** ^1^ Division of Physics Mathematics and Astronomy California Institute of Technology Pasadena CA 91125 USA; ^2^ Department of Advanced Materials Science University of Tokyo Kashiwa 277‐8561 Japan; ^3^ Centro Atomico Bariloche Instituto de Nanociencia y Nanotecnologia (CNEA‐CONICET) and Instituto Balseiro Av. Bustillo 9500 Bariloche Argentina; ^4^ RIKEN Center for Emergent Matter Science (CEMS) Wako 351‐0198 Japan; ^5^ Institute for Solid State Physics University of Tokyo Kashiwa Chiba 277‐8581 Japan; ^6^ Institute for Materials Research Tohoku University Sendai Miyagi 980‐8577 Japan; ^7^ National High Magnetic Field Lab Tallahassee FL 32310 USA; ^8^ Quantum‐Phase Electronics Center and Department of Applied Physics University of Tokyo Bunkyo‐ku Tokyo 113‐8656 Japan; ^9^ Photon Factory Institute of Materials Structure Science High Energy Accelerator Research Organization (KEK) Tsukuba 305‐0801 Japan; ^10^ Japan Synchrotron Radiation Research Institute Sayo 679‐5198 Japan; ^11^ Present address: Department of Advanced Materials Science University of Tokyo Kashiwa 277‐8561 Japan; ^12^ Present address: Advanced Science Research Center Japan Atomic Energy Agency Ibaraki Tokai 319‐1195 Japan; ^13^ Present address: Graduate School of Informatics and Engineering The University of Electro-Communications 1‐5‐1 Chofugaoka Chofu Tokyo 182‐8585 Japan

**Keywords:** anomalous hall effect, Kramers nodal‐line semimetal, quantum oscillations

## Abstract

Nodal‐line semimetals are a class of topological materials hosting one dimensional lines of band degeneracy. Kramers nodal‐line (KNL) metals/semimetals have recently been theoretically recognized as a class of topological states inherent to all non‐centrosymmetric achiral crystal lattices. The electronic structure of candidate KNL semimetal YAuGe is investigated by angle‐resolved photoemission spectroscopy (ARPES) and quantum oscillations as well as by density functional theory (DFT) calculations. DFT has revealed that YAuGe hosts KNLs on the Γ‐A‐L‐M plane of the Brillouin zone, that are protected by the time reversal and mirror‐inversion symmetries. Through ARPES and quantum oscillations, signatures of hole bands enclosing the Γ point are identified, and the observed splitting of quantum oscillation frequency with angle is attributed to spin‐orbit‐coupling‐induced band splitting away from the KNLs. Furthermore, it is shown that the degeneracy of the nodal lines along the Γ‐A line is lifted by the time‐reversal‐symmetry breaking when the Y is substituted by magnetic *R* ions (*R* = rare earth). This becomes a source of Berry curvature and contributes to the anomalous Hall effect in magnetic *R*AuGe. These findings establish *R*AuGe as a new class of KNL semimetals offering significant potential for engineering of anomalous magnetotransport properties via magnetic rare‐earth substitution.

## Graphical abstract

The rare‐earth intermetallics YAuGe is identified as a Kramers nodal‐line semimetal, a topological state inherent to non‐centrosymmetric achiral lattices. Magnetic rare‐earth substitution induces an anomalous Hall effect, highlighting the impact of time‐reversal symmetry breaking on topological electronic states and offering insights into designing the functionalities of nodal‐line semimetals.

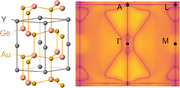

## 1 Introduction

Nodal‐line semimetals, where electron band crossings form one‐dimensional lines in the momentum space, offer a platform for exploration of a variety of novel phenomena, such as drumhead surface states as well as of enhanced quantum transport and optical responses.^[^ ^1^, ^2^, ^3^, ^4^ ^]^ Nodal lines have played a key role to understand the existence of Weyl points in a number of materials. In this context, nodal lines characterize the touching of valence and conduction bands in the absence of spin‐orbit interaction (SOI) and become split in the relativistic case.^[^ ^3^ ^]^ More recently, Kramers nodal‐line (KNL) semimetals were introduced in ref. [5], where it was shown that all time‐reversal symmetric noncentrosymmetric achiral materials are expected to host well‐defined nodal lines, even in the presence of strong SOI. These KNLs are protected by time‐reversal symmetry together with an achiral symmetry, such as a reflection symmetry. As a result, the degenerate states form lines that connect time‐reversal invariant momenta (TRIMs) of the Brillouin zone (BZ). A number of materials such as *T*RuSi (*T* = Ti, Nb, Hf, Ta),^[^ ^6^ ^]^ *R*Te_3_ (*R* = La, Y),^[^ ^7^, ^8^ ^]^ and SmAlSi^[^ ^9^ ^]^ are reported to host KNL from photoemission spectroscopy, while clear transport signatures associated with the KNL have not been well established yet.

**Figure 1:**
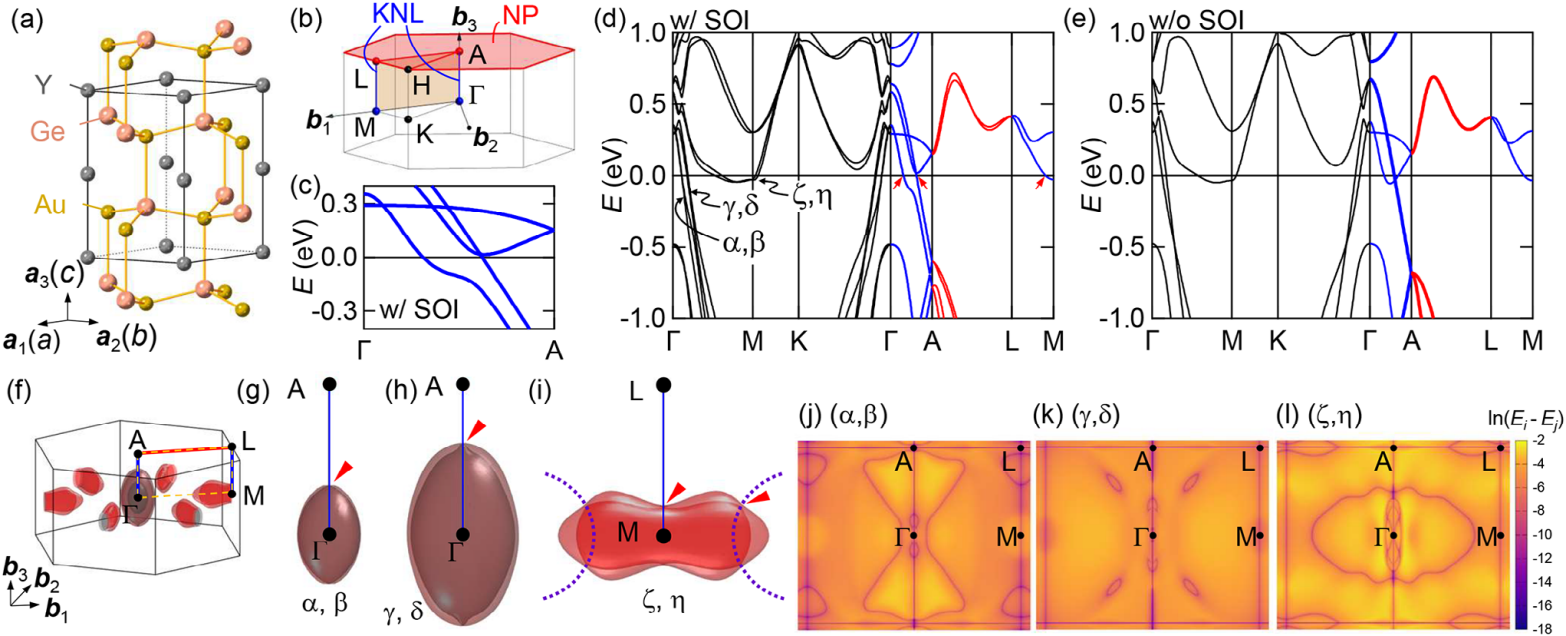
a) Crystal structure of YAuGe. b) BZ and high symmetry points. Γ, A, M, and L are TRIMs. Blue lines represent the KNLs, red hexagonal plane (*k*_*z*_ = π/*c*) is the NP, and orange area is invariant under the mirror inversion (*m*_010_). c) Band dispersion along the Γ‐A line in YAuGe, calculated with the SOI. All the bands are doubly degenerate KNLs. d,e) DFT calculations of the band structure in YAuGe (d) with SOI and (e) without SOI. In (d), bands along Γ‐A and M‐L lines form KNLs as denoted by blue curves, and red curves along A‐L line are twofold degenerate due to the NP nature. α, β, γ, and δ provide hole pockets at Γ, and ζ and η are electron pockets at M. Red arrows denote the position of the pinch point in FSs. In (e), thick curves along Γ‐A and A‐L lines denote fourfold degenerate bands. f) Fermi surfaces of YAuGe obtained by DFT calculations. Gray (red) sheets are for the hole (electron) pockets. g–i) Side view of the Fermi surfaces for α and β (γ and δ, ζ and η) branches. Blue line (red triangle) denotes KNL (pinch point). For (i), accidental NLs are also denoted by dashed purple curves, which gives additional four pinch points between ζ and η. j–l) Color map of the band energy difference plotted in a log scale, i.e., ln |*E*_*i*_ − *E*_*j*_| between j) *i* = α, *j* = β, k) *i* = γ, *j* = δ, and l) *i* = ζ, *j* = η. Purple curves correspond to the NLs.

*R*AuGe (*R* = Sc, La–Nd, Sm, Gd–Tm, and Lu) is a family of the *RTX* intermetallic phases (*T* is a transition metal element while *X* a *p*‐group element) belonging to the polar achiral structure (space group: *P*6_3_ *mc*) as shown in **Figure** **1**a,^[^ ^10^, ^11^ ^]^ satisfying the conditions to host KNLs. Au and Ge atoms form a wurtzite‐type network which breaks inversion symmetry, and *R* atoms form a triangular lattice in the *ab* plane. The interplay between the polar nature and high electrical conductivity of epitaxially grown thin films has recently attracted attention,^[^ ^12^, ^13^, ^14^ ^]^ and it has been reported that the introduction of magnetic ions in the *R* sites induces a large anomalous Hall effect.^[^ ^15^, ^16^ ^]^ The connection between these transport properties and the geometrical/topological characteristics of the electronic structure has not yet been studied to date.

Here, we investigate the electronic structure of polar YAuGe via a combination of ab initio density functional theory (DFT) calculations, angule‐resolved photoemission spectroscopy (ARPES), quantum oscillations in resistivity and magnetization torque, and specific heat. DFT calculations reveal that the electron bands near Fermi energy (*E*_F_) are of KNL nature along the Γ‐A line. In ARPES, we identify hole pockets centered at the Γ point, consistent with the DFT calculations and the dominant hole carrier transport observed in ref. [16]. The highly mobile hole pockets exhibit quantum oscillations at high magnetic fields, and an observed splitting with angle is attributed to due to the underlying spin‐orbit coupling in the system. Substitution of magnetic *R* atoms into Y site induces time‐reversal symmetry breaking, which lifts the degeneracy of KNL along the Γ‐A line. This effect gives rise to the finite Berry curvature among adjacent bands associated with the KNL. By comparing the experiments in *R*AuGe (*R* = Dy, Ho, Er, Tm) with DFT calculations, we identified that both extrinsic and intrinsic mechanisms contribute to the observed AHE. These findings demonstrate the presence of KNLs near the Fermi level in noncentrosymmetric *R*AuGe, and highlight their interplay with magnetic orders and time reversal symmetry.

## 2 Results

### 2.1 Density Functional Theory Calculations of Electronic Band Structure of YAuGe

We first discuss the KNL expected in YAuGe from the perspective of symmetry. Figure 1(b) represents the first BZ of YAuGe. The TRIMs are located at Γ, A, L, M, which are expected to be connected by the KNLs.^[^ ^5^ ^]^ Due to the mirror‐inversion symmetry, the KNLs are predicted to be confined on the Γ‐A‐L‐M plane (orange plane in Figure 1b).^[^ ^5^ ^]^ We note that the 6_3_ screw along the *c* axis leads to an additional degeneracy (nodal plane, NP) on the BZ boundary plane at *k*_*z*_ = π/*c* (red plane in Figure 1b).^[^ ^17^, ^18^, ^19^ ^]^ Symmetry arguments based on the representation theory are given in Supplementary Information A.

The above symmetry consideration is well consistent with the calculated band structure of YAuGe. Figure 1c shows the band structure with SOI along the Γ‐A line near *E*_F_. We note that all the shown bands are doubly degenerate and that the degeneracy cannot be lifted by SOI, as expected for KNL. Figure 1d,e compares the band structures with and without the SOI along high symmetry lines in the BZ highlighted in Figure 1b. Along Γ‐A, L‐M, and A‐L lines, the four‐fold degeneracy is lifted by the introduction of the SOI, while the twofold spin degeneracy is preserved, consistent with the symmetry considerations (see Supporting Information A). In contrast, the twofold spin degeneracy is fully lifted along Γ‐M, and Γ‐K lines. Without SOI, near Γ one can find two branches of doubly degenerate hole bands (Figure 1e) near *E*_F_; upon the introduction of SOI (Figure 1d), the noncentrosymmetric nature of the lattice splits each hole branch into two to form pairs of bands we assign as (α, β), and (γ, δ), respectively, while the degeneracy at Γ remains as the KNL runs through Γ. The same occurs around the M point, where the electron bands split into the (ζ, η) pair.

The Fermi surfaces of KNL semimetals are predicted to host the so‐called pinch points when a KNL crosses the Fermi level.^[^ ^5^ ^]^ At such points, two spin‐orbit‐split FSs touch with each other as denoted by red arrows in Figure 1d. Figure 1f–i depicts the FSs in YAuGe. The hole‐type FSs for the (α, β) and (γ, δ) pairs (Figure 1g,h) have two pinch points along Γ‐A (see red triangle). The electron pocket pair of (ζ, η) as shown in Figure 1i also has pinch points in the M‐L line connecting TRIMs, while one can identify four additional pinch points, as a result of accidental degeneracies near the Fermi level represented by purple dashed lines in Figure 1i.

To clarify the trajectory of KNLs in the BZ, the energy difference of SOI‐pair bands is plotted in the Γ‐A‐L‐M plane (Figure 1j–l). As predicted by the symmetry, the KNLs are observed as straight lines connecting along Γ‐A, and M‐L lines. We identify other branches of nodal lines forming self‐connected loops on the mirror plane, which are related to the additional pinch points of the (ζ, η) FSs. Such accidental NLs are allowed when the bands belong to different IRs, and the NLs are confined at the Γ‐M‐L‐A plane due to the same mechanism for the KNLs.^[^ ^5^ ^]^ Although this is an interesting aspect of the electronic structure hosting KNLs, in principle, these loops are not classified as the KNL because they do not connect the TRIMs.

### 2.2 Angle‐Resolved Photoemission Spectroscopy and Quantum Oscillation Measurements of YAuGe

**Figure 2:**
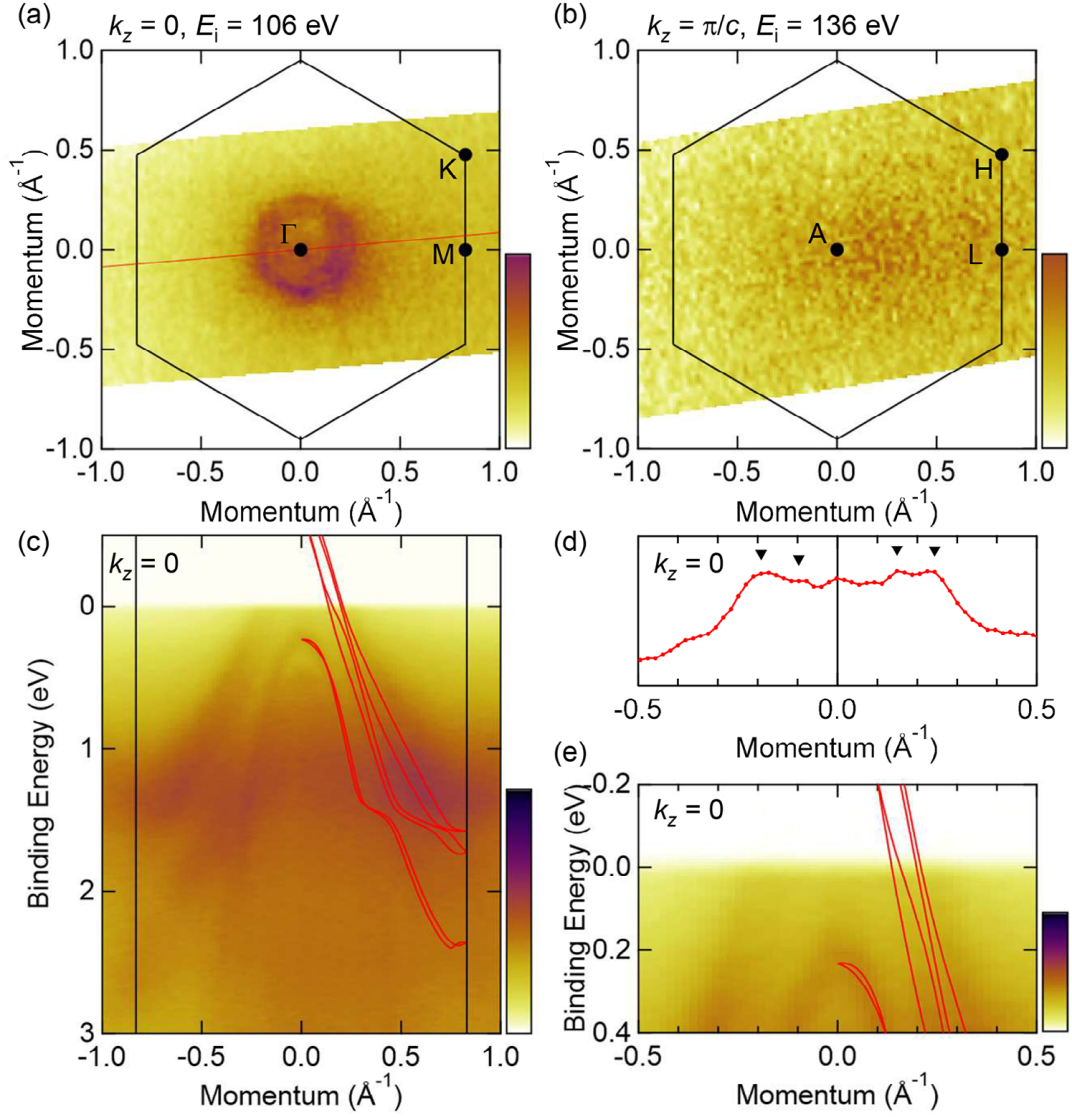
a,b) ARPES intensity plots at *E*_F_ of YAuGe measured at 15 K with *E*_i_ = 106 eV [136 eV] incident photons, which approximately probes the Γ‐M‐K [A‐L‐H] plane. The (001) surface BZ is marked with the black solid hexagon. c) Band dispersion along the momentum cut marked by the red line in (a) (near M‐Γ‐M). d) Intensity profile at *E*_F_ extracted from the ARPES image (c). Black triangles mark the intensity peaks for the hole bands. e) Zoom‐in image of the band dispersion at *E*_F_ for (c).

Having confirmed via DFT calculations the presence of KNLs near *E*_F_ in YAuGe, we investigate the electronic structure by ARPES. **Figure** **2**a,b shows the FS cross section collected at *T* = 15 K using incident photons of *E*_i_ = 106 eV and 136 eV, respectively, probing around the *k*_*z*_ = 0 and *k*_*z*_ = π/*c* planes. We find isotropic Fermi pockets near the Γ point in the *k*_*z*_ = 0 plane. The details of photon‐energy‐dependent ARPES results are provided in Supporting Information B. Figure 2c shows the band dispersion near the Γ‐M lines as depicted by the red line cut in Figure 2a. We have identified hole pockets from the spectra, which agrees well with the energy dispersion calculated by the DFT calculations (red curves). Here, we shifted *E*_F_ of the DFT calculation by –0.25 eV to overlap the band dispersions with the ARPES image. Considering that the Fermi momenta obtained by DFT calculation agree well with SdH measurement (as discussed below and summarized in Table S3, Supporting Information), this ARPES result might be suggesting the possible electron depletion occurring near the sub‐surface region of the cleaved crystal. Focusing on the bands near *E*_F_ (Figure 2d,e), we can identify signatures of two branches of bands that can be attributed to the two groups (α, β) and (γ, δ), while the SOI splitting within each pair appears to fall below experimental resolution.

**Figure 3:**
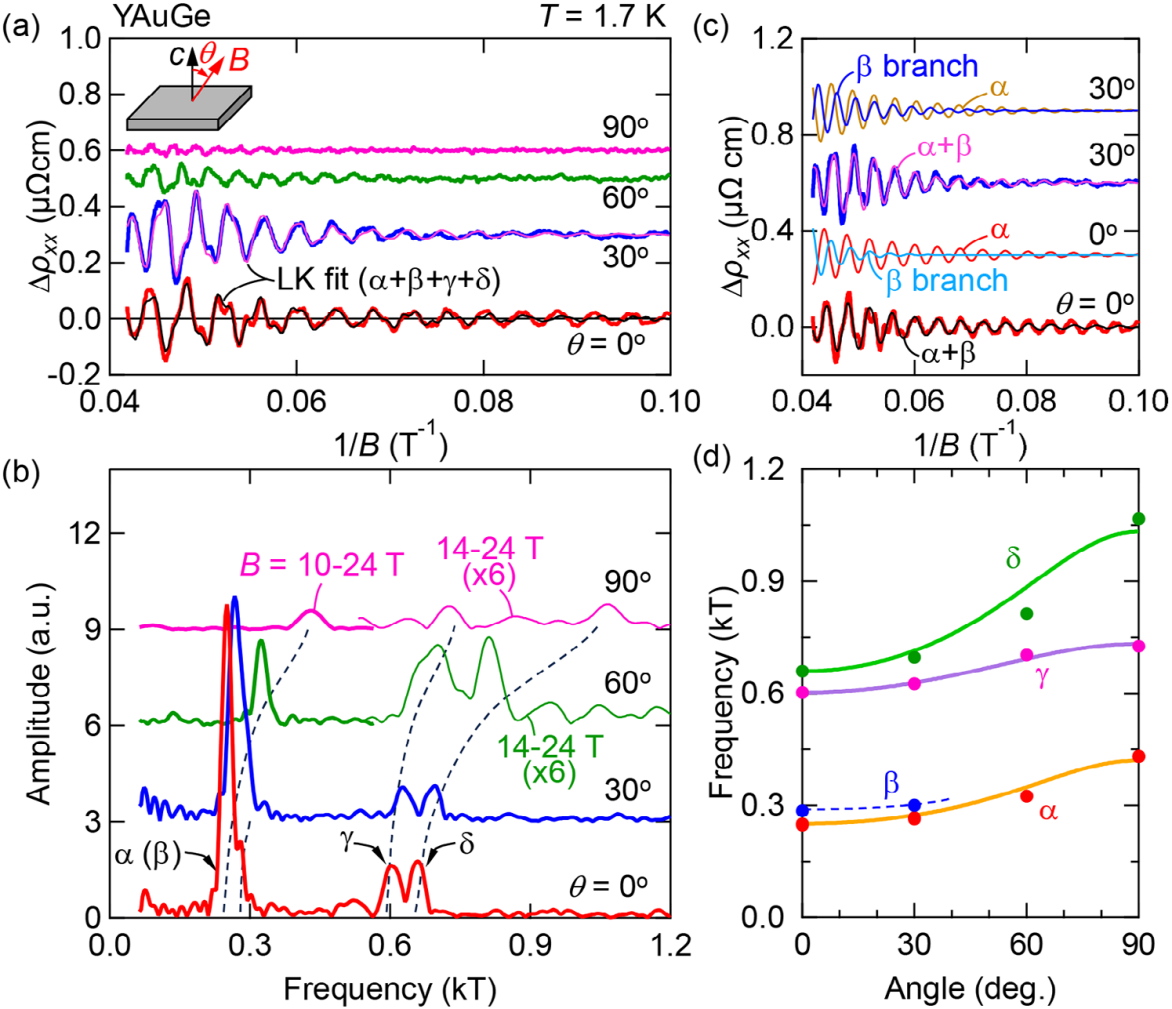
a) Inverse‐field (1/*B*) dependence of the background‐subtracted resistivity (Δρ_*xx*_) measured at *T* = 1.7 K with the *B* tilted from the *c* axis to the *ab* plane by θ (see inset). Thin black (pink) curve is the LK fit using Equation (1), where four branches (from α to δ) are considered. b) FFT of SdH oscillations at *T* = 1.7 K with various θ analyzed with different *B* windows. Thick: *B* = 10 to 24 T; thin: *B* = 14 to 24 T. Positions of the branches α, γ, and δ are assigned. The β branch is not resolved from the peak for α. Dashed curve is the guide to eyes. c) Comparison between SdH oscillations at θ = 0, 30° and the LK fit. For θ = 0°, thin black curve is the summation of the oscillation components for α and β and thin red (cyan) curve above is the individual oscillation components for α (β). For θ = 30°, thin pink curve is the summation of the oscillation components for α and β and thin yellow (blue) curve above is the individual oscillation components for α (β). d) Angular (θ) dependence of the SdH oscillation frequency for each branch (closed circles). Solid (dashed) lines are fits with the model of an elliptical Fermi surface (guide to eyes).

To better resolve the SOI splittings in the Fermi surfaces, we turn to Shubnikov‐de Haas oscillations in the resistivity of YAuGe at a high magnetic field up to 24 T. **Figure** **3**a shows quantum oscillations in Δρ_*xx*_ after subtraction of a smooth background. Fast Fourier transformation (FFT) of Δρ_*xx*_ reveals the oscillation frequencies as peaks (Figure 3b). At θ = 0, compared with the DFT calculations, we identify three branches α (*F*_α_ = 250 T), γ (*F*_γ_ = 602 T), and δ (*F*_δ_ = 659 T), while the expected peak for β overlaps with that of α. The temperature dependence of oscillation amplitude is analyzed with the Lifshitz–Kosevich (LK) formula (see Supporting Information C), which gives the effective mass: $m_{\alpha }^{*}=0.10m_{0}$, $m_{\gamma }^{*}=0.21m_{0}$, $m_{\delta }^{*}=0.25m_{0}$, where *m*_0_ is the free electron mass. The consistency with the DFT calculations is also confirmed (see Figure S4, Supporting Information).

As magnetic field is tilted from the *c* axis toward the *ab* plane, all the oscillation peaks shift to higher frequencies (Figure 3b), suggesting that the FSs are all elliptically elongated along the *k*_*z*_ direction. We note that the oscillations associated with the electron pockets around M (ζ and η) are not observed at any field angles possibly due to the large effective mass, which can also be inferred from the band dispersion in Figure 1d (see also Figure S4b, Supporting Information). From specific heat (see Supporting Information C), we estimate the effective mass of electron pockets to be $m_{\zeta ,\eta }^{*}\approx 0.4m_{0}$. We note that this is the averaged value of the effective mass tensor anisotropic in the momentum space, and the heavier mass than that for hole bands is consistent with DFT. We also note that in the previous study by some of the authors^[^ ^16^ ^]^ the electron carrier mobility was estimated to be around µ_*e*_ = 100 cm^2^V^−1^s^−1^ by a two‐carrier analysis, which requires *B* ≈ 100 T to resolve quantum oscillations (µ_*e*_ *B* ≈ 1).

Although not clearly resolved in the FFT spectra shown in Figure 3b, the coexistence of the α and β branches is indicated by the nonmonotonic evolution of oscillation amplitudes as a function of inverse field (see θ = 0° and θ = 30° in Figure 3a), suggesting a beating pattern between the closely spaced α and β branches. In order to resolve these two branches, we fit the Δρ_*xx*_ vs 1/*B* with the LK formula as below^[^ ^20^ ^]^

(1) $$\Delta \rho_{xx}=\sum_{i=\alpha ,\text{…}}\sum_{p=1,\text{…}}N_{i,p}B^{1/2}R_{T}^{i,p}R_{D}^{i,p}\cos2\pi (pF_{i}/B+\phi_{i,p})$$

where *N*_*i*, *p*_ is oscillation amplitude, *F*_*i*_ is the oscillation frequency, ϕ_*i*, *p*_ is the phase shift. The temperature damping factor *R*_*T*_ for *p* = 1 is given in Equation S1 (Supporting Information). The Dingle damping factor is given by

(2) $$R_{D}=\exp\left(-\frac{2\pi^{2}k_{B}T_{D}m^{*}}{\hbar eB}\right)$$

where *T*_D_ is the Dingle temperature. The spin reduction factor $R_{S}=\cos\left(\frac{p\pi g^{*}m^{*}}{2m_{0}}\right)$ with an effective g* factor is not included because this is only applicable to the system with both time‐reversal and spatial‐inversion symmetries. Phase shift ϕ for the *p*‐th harmonics is given by

(3) $$\phi_{p}=-p/2+\left(\phi_{B}+\phi_{R}+\phi_{Z}\right)/2\pi +\phi_{3D}+\phi_{Z}^{\prime }$$

where ϕ_B_ is the Berry phase (0 and π for trivial and nontrivial Fermi surface, respectively),^[^ ^21^ ^]^ ϕ_R_ is the orbital moment, ϕ_Z_ is the spin Zeeman effect, and ϕ_3D_ = ±1/8 for 3D Fermi surface or ≈ ± 0 for quasi‐2D Fermi surface (+: hole, and −: electron pocket).^[^ ^20^, ^22^ ^]^ $\phi_{s}^{\prime }$ is the field‐dependent correction related to the magnetic susceptibility.^[^ ^23^, ^24^, ^25^ ^]^

The branches α and β (δ and η) are originated from the spin‐orbit splitting bands. In such a case, ϕ_B_ is expected to be the nontrivial π,^[^ ^5^, ^21^ ^]^ while ϕ_R_ and ϕ_Z_ average out for the orbit that encircles the TRIM (Γ point).^[^ ^24^, ^26^, ^27^ ^]^ Although the quality of the experimental data is not sufficient to unambiguously resolve ϕ_B_ + ϕ_R_ + ϕ_Z_ by fitting, we set ϕ_B_ = π and ϕ_R_ + ϕ_Z_ = 0 to reduce the number of free parameters for simplicity. We treat $\phi_{s}^{\prime }$ as a perturbation to be linear in *B*, exhibiting opposite signs between α and β (and γ and δ). This corresponds to the adiabatic limit or a weak field limit,^[^ ^25^ ^]^ where the Zeeman effect is small compared to the spin‐orbit splitting. The fitting results are consistent with this approximation when the *B* is up to 24 T. We also note that the frequency splitting between α and β remains robust regardless of the inclusion or omission of $\phi_{s}^{\prime }$. Since all the oscillation branches from α to δ are hole pockets with ellipsoidal shape, we set ϕ_3D_ = +1/8.^[^ ^22^ ^]^

The best fit is given by thin curves in Figure 3a. As the *R*_*T*_ and *R*_D_ interfere with each other, m* in *R*_*T*_ and *R*_D_ is fixed at the value obtained by the mass plot analysis. Figure 3c shows the extracted individual oscillation components for α and β branches along with their summation, and the latter well reproduces the beating of the main oscillations for both angles. Figure 3d summarizes the angular dependence of oscillation frequencies for all the four observed branches, which is in good agreement with DFT calculations (Figure S4, Supporting Information).

We also perform magnetization torque (τ) measurements with the pulse field up to 60 T and observe de Haas–van Alphen oscillations (see Figures S6 and S7, Supporting Information). Similar with our analysis above for the Shubnikov‐de Haas oscillations, we identify four hole branches through a combination of FFT and the fitting of the LK formula to the τ vs 1/*B* curves. The oscillation frequencies, effective mass, and angular dependence of frequencies are consistent with the analysis of the SdH oscillations (see Table S3, Supporting Information).

The splittings of the quantum oscillations for (α, β) and (γ, δ) are a signature of SOI, which has been identified in various noncentrosymmetric systems.^[^ ^28^, ^29^ ^]^ In the approximation of the Rashba model given the polar symmetry of YAuGe, the effect of the SOI is estimated by the splitting of the band energy

(4) $$\varepsilon_{p\pm }=\frac{p^{2}}{2m^{*}}\mp \alpha p_{⊥}$$

where $p_{⊥}=\sqrt{p_{x}^{2}+p_{y}^{2}}$, m* is the effective mass, and α is the coefficient of SOI. The energy splitting (Δϵ = 2α*p*_F_) is related to the frequency splitting (Δ*F*) as

(5) $$\Delta \varepsilon =\frac{\hbar e\Delta F}{m^{*}}$$

The magnitude of Δϵ in YAuGe is estimated to be 45 meV (for Δ*F* = *F*_β_ − *F*_α_) and 27 meV (for Δ*F* = *F*_δ_ − *F*_γ_), the order of magnitude of which is comparable with typical polar achiral rare‐earth transition‐metal tetrel compounds (Δϵ ∼ 10 − 100 meV).^[^ ^30^, ^31^, ^32^, ^33^ ^]^

### 2.3 Anomalous Hall Effect in Magnetic *R*AuGe

A large anomalous Hall conductivity has been reported in *R*AuGe with magnetic 4f electrons at *R* site.^[^ ^15^, ^16^ ^]^ The KNL in a polar system like YAuGe may be viewed as a three‐dimensional generalization of the crossing point in the two‐dimensional Rashba band accumulated along the Γ‐A line. The interplay between 2D Rashba models and time‐reversal symmetry breaking is well studied theoretically, where time reversal symmetry breaking is expected to lift the degeneracy and generate significant Berry curvatures near the gapped crossing point.^[^ ^34^, ^35^, ^36^, ^37^ ^]^ This in principle leads to concentrated Berry curvature distributions along the entire gapped nodal line, and such scenario can also be closely compared to the role of gapped nodal lines by ferromagnetic order and spin‐orbit coupling near K points to anomalous Hall effect discussed for Fe_3_GeTe_2_ and Fe_3_Sn_2_.^[^ ^38^, ^39^ ^]^ As a last part of this paper, we discuss the connection between the anomalous transport properties of magnetic *R*AuGe and the KNLs.

A simple picture to understand the emergence of AHE in magnetic *R*AuGe is that magnetic moments break the time‐reversal symmetry of the Rashba‐split bands. In the rigid band approximation, the substitution of *R* ions with Y ion does not significantly change the electronic bands besides the exchange field from the localized 4f electrons. This is a reasonable assumption for *R* = Dy‐Tm since the 4f electrons are deep below the Fermi level, i.e., band bending due to c‐f hybridization near the Fermi level is negligible.

**Figure 4:**
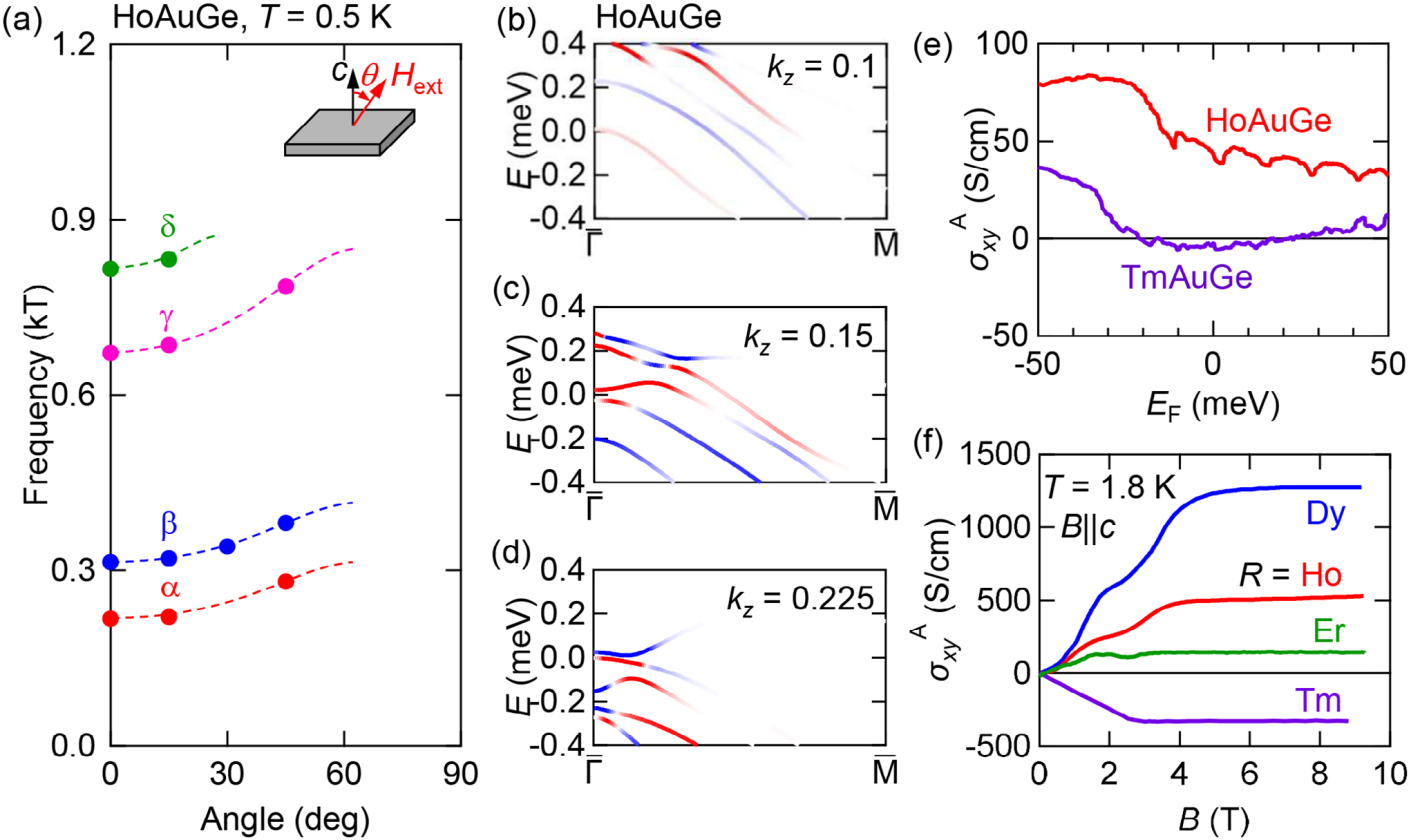
a) Angular (θ) dependence of the SdH oscillation frequency for each branch (closed circles) observed at *T* = 0.5 K in HoAuGe. Dashed lines are guide to eyes. b–d) Berry curvature in HoAuGe at different *k*_*z*_. e) AHC ($\sigma_{xy}^{A}$) for HoAuGe and TmAuGe calculated with open core (see methods). f) Magnetic field dependence of AHC observed in *R*AuGe (*R* = Dy‐Tm) for *B*∥*c* at *T* = 1.8 K. For *R* = Dy and Ho, $\sigma_{xy}^{A}$ is reproduced from ref. [16] with permission.

To analyze if these considerations are reasonable, we measure the SdH oscillation in HoAuGe up to 31 T. As discussed in details in Supplementary Information D, we have identified four oscillation branches with frequencies close to those observed in nonmagnetic YAuGe (see Figure S8, Supporting Information). The estimated effective mass is also comparable to those for YAuGe, indicating that the itinerant electronic bands do not strongly deviate from that of the non‐magnetic YAuGe. **Figure** **4**a summarizes the angular dependence of SdH oscillation frequencies. We note that the splitting between α and β (γ and η) in HoAuGe is enhanced from that in YAuGe, which may be due to the exchange interaction between the localized Ho 4f electrons and conduction electrons.

To capture the effect of time‐reversal symmetry breaking, we calculated the band structure in ferromagnetic HoAuGe. These calculations are done treating the 4f‐shell in the open‐core approximation. Figure 4b–d shows the band structure in the ferromagnetic state with the magnetic moment at Ho^3 +^ pointing along the *c* axis. The degeneracy at the Γ point protected by time‐reversal symmetry in YAuGe is lifted by the magnetic moments which causes the emergence of net Berry curvature.

The anomalous Hall conductivity (AHC, $\sigma_{xy}^{A}$) as a function of *E*_F_ is calculated for ferromagnetic states of HoAuGe and TmAuGe and summarized in Figure 4e. As illustrated for the case of HoAuGe in Figure 4b–d, the contributions to the intrinsic anomalous Hall conductivities is concentrated near the gapped KNL. The *E*_F_ dependence of $\sigma_{xy}^{A}$ is shown in Figure 4e. Near *E*_F_, HoAuGe has a finite positive AHC, which is in contrast to a small negative value in TmAuGe. The observed AHC shows the *R* dependence as it systematically decreases for *R* = Dy, Ho, and Er and changes its sign at *R* = Tm (Figure 4e, see ref. [16] and Supporting Information D for the detailed analysis). We note, however, that the magnitude of the Hall conductivity at *E*_F_ for both compounds is much smaller than the observed AHC.

One possibility for this discrepancy is that the dominant contribution of the measured AHC stems from extrinsic mechanisms such as skew scattering.^[^ ^36^, ^37^ ^]^ It is known that the extrinsic mechanism dominates the AHE when the system is in the clean regime, which is obtained at *E*_F_τ/ℏ > >π/2, where τ is the relaxation time. By a rough estimate of $E_{F}\left(=\frac{\hbar^{2}k^{2}F}{2m_{\text{eff}}}\right)$ and $\tau (=\frac{\mu m_{\text{eff}}}{e})$, we obtain *E*_F_τ/ℏ ≈ 20 − 50 for the hole pockets in HoAuGe. This suggests that the current system may very well be on the verge of the crossover from the intrinsic to extrinsic regime, and hosts a significant extrinsic contribution to the AHE. A second possibility is that the adopted open‐core approximation is not appropriate to quantitatively describe the low‐energy electronic structure. Although we have shown that the quantum oscillation patterns and associated effective masses do not significantly change between YAuGe and HoAuGe, the Berry curvature relevant to the AHC depends strongly on the precise gap sizes induced by the long‐range order in the KNLs, and these may in turn be affected by hybridization between the local moments and conduction electrons which are neglected in the open‐core approximation. Further investigation such as doping between magnetic and non‐magnetic *R*AuGe and comparing their transport properties may shed additional light on the anomalous magnetotransport responses in KNL semimetals.

## 3 Conclusion

In conclusion, we have revealed the electronic structure of a KNL semimetal candidate YAuGe, and established the existence of KNLs by symmetry considerations and DFT calculations. The high‐mobile hole carriers originated from KNLs crossing the Fermi level are identified by ARPES and quantum oscillations. Additionally, the spin splitting of these bands—‐a hallmark of inversion symmetry breaking in the lattice—has been resolved through quantum oscillation studies. We also discussed the anomalous magnetotransport properties observed in *R*AuGe, suggesting that both intrinsic and extrinsic mechanisms may contribute, with the intrinsic effects arising from the interplay between time‐reversal symmetry breaking and the KNLs. These insights highlight the potential of KNL semimetals as platforms for exploring spin‐orbit coupling‐induced spin splitting and enhanced anomalous magnetotransport properties when broken time‐reversal symmetry is incorporated.

## Conflict of Interest

The authors declare no conflict of interest.

## Supporting information

Supporting Information

## Data Availability

The data that support the findings of this study are available from the corresponding author upon reasonable request.
