# Supplementary material for: Electronic Structure of Kramers Nodal‐Line Semimetal YAuGe and Anomalous Hall Effect Induced by Magnetic Rare‐Earth Substitution
Source: Adv Sci (Weinh). 2025 May 8;12(27):2501669. doi: 10.1002/advs.202501669 (PMC12279210; doi:10.1002/advs.202501669)
Supplement: Supplementary file 1 — Supporting Information [file ADVS-12-2501669-s001.pdf]

ADVANCED SCIENCE
Open Access

Supporting Information

for *Adv. Sci.*, DOI 10.1002/advs.202501669

Electronic Structure of Kramers Nodal-Line Semimetal YAuGe and Anomalous Hall Effect Induced by Magnetic Rare-Earth Substitution

Takashi Kurumaji, Jorge I. Facio, Natsuki Mitsuishi, Shusaku Imajo, Masaki Gen, Motoi Kimata, Linda Ye, David Graf, Masato Sakano, Miho Kitamura, Kohei Yamagami, Kyoko Ishizaka, Koichi Kindo and Taka-hisa Arima*

Supporting Information for "Electronic structure of the Kramers nodal-line semimetal YAuGe and anomalous Hall effect induced by magnetic rare-earth substitution"

Takashi Kurumaji *et al.*

A. SYMMETRY ANALYSIS AND DENSITY FUNCTIONAL THEORY CALCULATIONS OF THE ELECTRONIC STRUCTURE OF YAUGE

The crystal structure of YAuGe belongs to the non-symmorphic noncentrosymmetric space group $P6_3mc$ (SG.186, C_{6v}^4). The primitive lattice vectors are $\boldsymbol{a}_1 = (0, -a, 0)$, $\boldsymbol{a}_2 = (\frac{\sqrt{3}a}{2}, \frac{a}{2}, 0)$, and $\boldsymbol{a}_3 = (0, 0, c)$, where the lattice constants are $a = 4.41$ Å, $c = 7.27$ Å, respectively, at $T = 30$ K [S1]. In a unit cell, there are two sets of formula unit, i.e., $Z = 2$ (see Fig. 1). The reciprocal lattice vectors are $\boldsymbol{b}_1 = \frac{2\pi}{a}(\frac{1}{\sqrt{3}}, -1, 0)$, $\boldsymbol{b}_2 = \frac{2\pi}{a}(\frac{2}{\sqrt{3}}, 0, 0)$, and $\boldsymbol{b}_3 = \frac{2\pi}{c}(0, 0, 1)$.

The degeneracy of electronic bands at high-symmetry points and along lines in BZ (Fig. S1) can be systematically analyzed by group theory [S2]. Tables S1 and S2 summarize the Herring's little groups ${}^{\mathrm{H}}G_{\boldsymbol{k}}(= G_{\boldsymbol{k}}/T_{\boldsymbol{k}})$ ($G_{\boldsymbol{k}}$ is the little group at momentum $\boldsymbol{k}$ and $T_{\boldsymbol{k}}$ is the group of translational symmetry operations $\{E|\boldsymbol{t}\}$ with $\exp(-\mathrm{i}\boldsymbol{k}\cdot\boldsymbol{t}) = 1$) for $P6_3mc$ and their single and double-valued irreducible representations (IRs).

TRIMs are located at Γ $(= (0, 0, 0))$, A $(= (0, 0, \frac{1}{2}))$, M $(= (0, \frac{1}{2}, 0))$, and L $(= (0, \frac{1}{2}, \frac{1}{2}))$ points, where the coordinates are taken by the reciprcal lattice vectors. The ΓALM-plane is invariant under a mirror inversion symmetry ($s_0 = \{m_{100}|000\}$, where the coordinates are taken by the primitive lattice vectors). Δ^x and U^x lines connecting Γ-A and M-L, respectively, are two-fold degenerate even with the SOI, which are identified as KNL. We note that A and L are four-fold degenerate even with the SOI. This is owing to 6_3-screw ($s_1 = \{C_6^+|00\frac{1}{2}\}$) and c-glide ($s_2 = \{m_{1\bar{1}0}|00\frac{1}{2}\}$) symmetries, respectively, of the lattice as pointed out in Ref. S3 and S4. Furthermore, the 6_3-screw makes the $k_z = \pi/c$-plane at the BZ boundary a nodal plane (NP) because the points upon this plane are invariant under the combination of time-reversal ($\mathcal{T}$) and the 6_3 screw ($s_1\mathcal{T}$) [S5]. We note that P^x (K-H) may have two-fold degenerate bands when their IR belong to $\bar{E}_1(2)$ (see Table S2), while this is beyond the scope of this study as the band energy at K-H line in YAuGe is sufficiently away from E_{F}.

The symmetry analysis above is consistent with the DFT calculation shown in Figs. 1(d)-(e). In the presence of SOI (Fig. 1(d)), the band energy has four-fold degeneracy at A and L points, and they split into two two-fold degenerate bands along Δ^x (Γ-A line), R^x (A-L line), U^x (M-L line), S^x (A-H line), and S'^x (L-H line) [S6]. Along Σ^x (Γ-M line) and T^x (Γ-K line), all the bands lift the degeneracy, which ensure the splitting of the bands from α to η at Fermi energy (E_{F}) in k_xk_y plane. Along the k_z direction, pairs of Fermi surfaces, i.e, α-β, γ-δ, and ζ-η, has a touching point due to the KNL nature. We note that the Fermi surface of ζ-η pair has other touching points as denoted in Fig. 1(i). By shifting E_{F}, the touching points draw loops in the BZ as shown in Fig. 1(l), which are pinned on the ΓALM plane by the s_0 symmetry [S7].

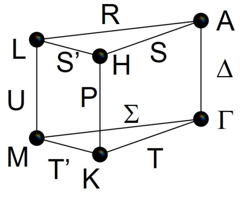

FIG. S1. Symbols of the high-symmetry points and lines in the BZ of YAuGe.

TABLE S1. The Herring's little group ${}^{\mathrm{H}}G^{\boldsymbol{k}} = G^{\boldsymbol{k}}/T^{\boldsymbol{k}}$ on points or lines of symmetry in the BZ of YAuGe, and the single-valued irreducible representations (IRs). The number in the parenthesis following each IR represents the dimension of the corresponding IR. The label $\leftrightarrow$ between two IRs means that these two IRs are paired up to form time-reversal-invariant representations (co-representations). The total number of degeneracy is obtained by doubling the number in parentheses, which is due to the spin degrees of freedom. The label convention is adopted as the same of Ref. S2.

	${}^{\mathrm{H}}G^{\boldsymbol{k}}$	Single-valued IRs
Point of symmetry		
Γ	G_{12}^{3}	$A_1(1)$, $A_2(1)$, $B_2(1)$, $B_1(1)$, $E_2(2)$, $E_1(2)$
M	$G_4^2 \otimes T_2$	$A_1(1)$, $A_2(1)$, $B_1(1)$, $B_2(1)$
A	G_{24}^{4}	${}^1E_1(1){\leftrightarrow}{}^2E_1(1)$, ${}^1E_2(1){\leftrightarrow}{}^2E_2(1)$, ${}^1F(2){\leftrightarrow}{}^2F(2)$
L	G_8^2	${}^2E'(1){\leftrightarrow}{}^1E'(1)$, ${}^2E''(1){\leftrightarrow}{}^1E''(1)$
K	$G_6^2 \otimes T_3$	$A_1(1)$, $A_2(1)$, $E(2)$
H	$G_{12}^4 \otimes T_3 \otimes T_2$	${}^1E_1(1){\leftrightarrow}{}^2E_1(1)$, $E_2(2)$
Line of symmetry		
Δ^x	G_{12}^{3}	$A_1(1)$, $A_2(1)$, $B_2(1)$, $B_1(1)$, $E_2(2)$, $E_1(2)$
U^x	G_4^2	$A_1(1)$, $A_2(1)$, $B_1(1)$, $B_2(1)$
P^x	G_6^2	$A_1(1)$, $A_2(1)$, $E(2)$
T^x	G_2^1	$A'(1)$, $A''(1)$
S^x	G_2^1	$A'(1){\leftrightarrow}A''(1)$
T'^x	G_2^1	$A'(1)$, $A''(1)$
S'^x	G_2^1	$A'(1){\leftrightarrow}A''(1)$
Σ^x	G_2^1	$A'(1)$, $A''(1)$
R^x	G_2^1	$A'(1)({\leftrightarrow}A'(1))$, $A''(1)({\leftrightarrow}A''(1))$

TABLE S2. The double group and double-valued irreducible representations of the quotient group ${}^{\mathrm{H}}G^{\boldsymbol{k}} = G^{\boldsymbol{k}}/T^{\boldsymbol{k}}$ on points or lines of symmetry in the BZ of YAuGe. The label convention is adopted as the same of Ref. S2.

	Double group of ${}^{\mathrm{H}}G^{\boldsymbol{k}}$	Double-valued IRs
Point of symmetry		
Γ	G_{24}^{11}	$\bar{E}_1(2)$, $\bar{E}_2(2)$, $\bar{E}_3(2)$
M	$G_8^5 \otimes T_2$	$\bar{E}(2)$
A	G_{48}^{13}	$\bar{E}(2)({\leftrightarrow}\bar{E}(2))$, ${}^1\bar{F}(2){\leftrightarrow}{}^2\bar{F}(2)$
L	G_{16}^{8}	$\bar{E}(2)({\leftrightarrow}\bar{E}(2))$
K	$G_{12}^4 \otimes T_3$	${}^1\bar{E}(1)$, ${}^2\bar{E}(1)$, $\bar{E}_1(2)$
H	$G_{12}^4 \otimes T_3 \otimes T_2$	$\bar{A}_1(1){\leftrightarrow}\bar{A}_2(1)$, $\bar{E}(2)$
Line of symmetry		
Δ^x	G_{24}^{11}	$\bar{E}_1(2)$, $\bar{E}_2(2)$, $\bar{E}_3(2)$
U^x	G_8^5	$\bar{E}(2)$
P^x	G_{12}^4	${}^1\bar{E}(1)$, ${}^2\bar{E}(1)$, $\bar{E}_1(2)$
T^x	G_4^1	${}^2\bar{E}(1)$,${}^1\bar{E}(1)$
S^x	G_4^1	${}^2\bar{E}(1){\leftrightarrow}{}^1\bar{E}(1)$
T'^x	G_4^1	${}^2\bar{E}(1)$,${}^1\bar{E}(1)$
S'^x	G_4^1	${}^2\bar{E}(1){\leftrightarrow}{}^1\bar{E}(1)$
Σ^x	G_4^1	${}^2\bar{E}(1)$,${}^1\bar{E}(1)$
R^x	G_4^1	${}^2\bar{E}(1)({\leftrightarrow}{}^2\bar{E}(1))$,${}^1\bar{E}(1)({\leftrightarrow}{}^1\bar{E}(1))$

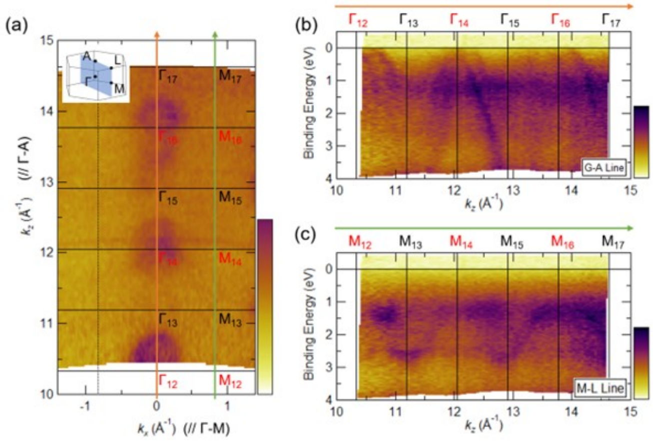

FIG. S2. (a) ARPES intensity plots at E_F [integral width: 50 meV] in the Γ-M-L-A plane for YAuGe, collected at $T = 20$ K using $E_i = 400$-800 eV incident photons. (b), (c) ARPES images along Γ-A and M-L lines (see orange/green lines in (a)).

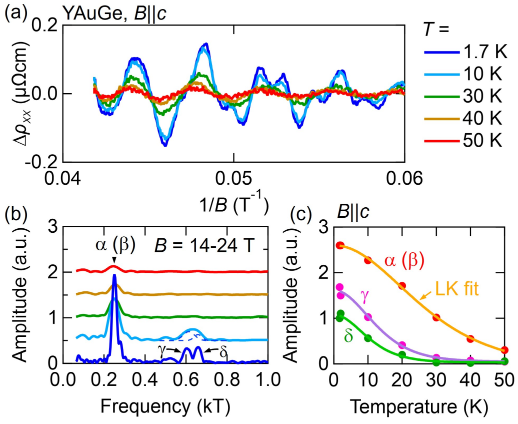

FIG. S3. (a) B-inverse field dependence of the background-subtracted resistivity ($\Delta\rho_{xx}$) measured at various temperatures with the B along the c axis. (b) FFT of SdH oscillations for $B \parallel c$ at various temperatures. Positions of the branches α, γ, and δ are assigned. The β branch is overlapped with α. Dashed curve is the gaussian functions that fit a broad peak for γ and δ pockets. (c) Temperature dependence of peak amplitude (closed circles) and fits with LK formula (solid lines) for each branch.

B. SOFT X-RAY ARPES EXPERIMENTS IN YAUGE

To investigate the three-dimensionality of band structures in YAuGe, we have performed photon-energy-dependent ARPES measurements in the soft x-ray photon energy regime. Figure S2(a) displays the ARPES intensity plots at E_F in the Γ-M-L-A plane (see inset), obtained by changing the incident photons from $E_i = 400$ to 800 eV with interval of 5 eV. For this soft x-ray dataset, we assume the inner potential of 20 eV to plausibly convert the photoelectron emission angle into momentum. We find the signatures of ellipse Fermi pockets centered at every even Γ points (such as Γ_{14} and Γ_{16}), but such intensity is mostly absent at odd Γ (Γ_{13} and Γ_{15}). This double periodicity (i.e., $4\pi/c$ periodicity) of ARPES spectra can also be observed in the band dispersion along Γ-A and M-L directions as shown in Fig. S2(b) and (c). Such feature is characteristic to materials with a nonsymmorphic space group, as known for example in $2H$-WSe_2[S8] and BiTeCl[S9].

C. QUANTUM OSCILLATIONS AND SPECIFIC HEAT IN YAUGE

A. Shubnikov-de Haas oscillations

To characterize the effective mass of each branch, we measure the SdH oscillations in YAuGe at various temperatures with $B \parallel c$ (Fig. S3(a)). As shown in Fig. S3(b), the FFT identifies the three branches, α, γ, and δ at the lowest temperatures, while the frequency for the β branch is overlapped with that of α. Increasing the temperature, the frequency peaks for γ and δ merge to a single peak. The γ and δ are resolved by a fit of the peak with two Gaussian functions at fixed frequencies observed at $T = 1.7$ K as shown by blue dashed curve for $T = 10$ K. The temperature dependence of each oscillation amplitude is shown in Fig. S3(c). From these data we estimate the effective mass by using the temperature damping factor in a Lifshitz-Kosevich (LK) formula as below [S10].

$$R_T = \frac{2\pi^2 p k_B T m^*}{\hbar e B} \sinh^{-1}\left(\frac{2\pi^2 p k_B T m^*}{\hbar e B}\right), \quad \text{(S1)}$$

where $\hbar$ is the Planck constant divided by 2π, k_B is the Boltzmann constant, $e(>0)$ is the elementary charge, m^* is the effective mass, p is the number of harmonics [S10]. B is replaced with the average $B_{av} = \{\frac{1}{2}(1/B_h + 1/B_l)\}^{-1}$ between the highest ($B_h = 24$ T) and lowest ($B_l = 14$ T) fields. The physical parameters of each branch are summarized in Table S3.

The angular dependence of oscillation frequency and the effective mass is simulated by DFT calculations. Figure S4(a) shows the frequencies of FS extrema for the plane perpendicular to the magnetic field. The frequencies for the hole pockets become higher as the field is tilted towards the ab plane. This is due to the elliptically elongeted FSs along the k_z directions (see Figs. 1(g)-(h)). Figure S4(b) is the angular dependence of the effective mass for each frequency branch. Those for the hole pockets are $0.1 \sim 0.2 m_e$, while the electron bands have relatively heavy mass.

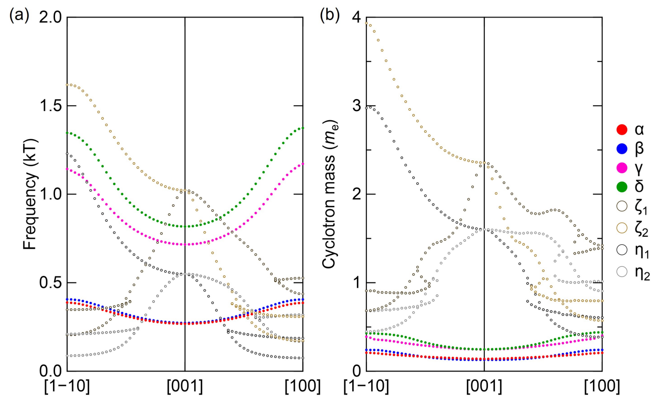

FIG. S4. The DFT calculations of (a)-(b) magnetic field orientation dependence of (a) quantum oscillation frequency and (b) effective mass for each branch.

B. Specific heat

To see the signature of the electron pockets, we have measured the temperature dependence of specific heat in YAuGe down to 0.5 K. As shown in Fig. S5, C_{p}/T vs. T^2 plot has a linear slope, which can be fit with

$$C_{\mathrm{p}}/T = \gamma_{\mathrm{c}} + \beta_{\mathrm{ph}} T^2, \tag{S2}$$

where γ_{c} and β_{ph} is the coefficient for conduction electrons and phonons, respectively. As a comparison, we also measured C_{p} for LuAuGe, where γ_{c} is almost identical, which is reasonable for the nonmagnetic sibling (isoelectronic and isostructural) compounds. The steeper T^2-evolution in LuAuGe, i.e., higher β_{ph}, is consistent with the Debye model, giving a lower Debye temperature due to the heavier molecular masses in LuAuGe than in YAuGe [S11,S12]. The Debye temperature (Θ_{D}) is obtained by $\Theta_{\mathrm{D}} = (\frac{12\pi^4 k_{\mathrm{B}} N_{\mathrm{A}} n}{5\beta_{\mathrm{ph}}})^{1/3}$, where n ($= 3$) is the number of atoms in a formula unit. We obtain $\Theta_{\mathrm{D,Y}} = 289$ K and $\Theta_{\mathrm{D,Lu}} = 247$ K. The coefficient γ_{c} is proportional to the density of states ($D(E_{\mathrm{F}})$) as

$$\gamma_{\mathrm{c}} = \frac{\pi^2}{3} k_{\mathrm{B}}^2 D(E_{\mathrm{F}}). \tag{S3}$$

The observed γ_{c} ($= 0.659$ mJ/mol K^2 in YAuGe, and 0.741 mJ/mol K^2 in LuAuGe) correspond to $D_{\mathrm{tot}} =$ 0.280 eV^{-1} f.u.$^{-1}$ and 0.314 eV^{-1} f.u.$^{-1}$, respectively

The contribution from the electron pockets (D_{e}) in YAuGe can be obtained by subtracting the hole components (D_{h}) from D_{tot}. We estimate the D_{h} from the SdH results, by assuming that the shape of the Fermi surfaces is elliptical with the long axis ($2k_{\mathrm{F}\parallel}$) along the c^* axis and short axis ($2k_{\mathrm{F}\perp}$) in the ab plane. This approximation is in good agreement with the angular dependence of the SdH oscillations (Fig. 3(d)). The density of states

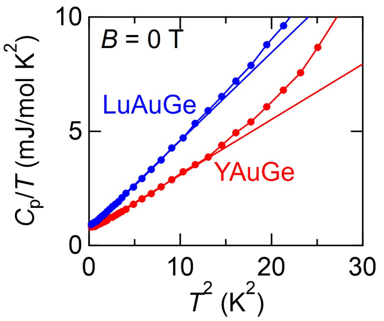

FIG. S5. Temperature dependence of zero field specific heat (C_{p}) for YAuGe and LuAuGe. Solid lines are the linear fit for the low-temperature slope.

(D_{ellip}) is given by

$$D_{\mathrm{ellip}} = \frac{1}{8\pi^3}\frac{\partial}{\partial E_{\mathrm{F}}}(\frac{4\pi}{3}k_{\mathrm{F}\parallel}k_{\mathrm{F}\perp}^2) = \frac{1}{6\pi^2\hbar^2}(2k_{\mathrm{F}\parallel}m_{\perp}^* + \frac{k_{\mathrm{F}\perp}^2 m_{\parallel}^*}{k_{\mathrm{F}\parallel}}), \tag{S4}$$

where $m_{\parallel,\perp}^*$ is the effective mass along c^* axis and the ab plane. By assuming $m_{\parallel,\perp}^*$ to be identical to the estimated effective masses for each band in $B \parallel c$ (Fig. S3(c)), we obtain $D_{\mathrm{h}} = D_\alpha + D_\beta + D_\gamma + D_\delta = 0.041$ eV^{-1} f.u.$^{-1}$. As a consequence, we obtain the $D_{\mathrm{e}} = D_{\mathrm{tot}} - D_{\mathrm{h}} = 3(D_\zeta + D_\eta) = 0.24$ eV^{-1} f.u.$^{-1}$. The factor three represents the valley degrees of freedom for the pocket at the M point. The dominant electron contribution to $D(E_{\mathrm{F}})$ is consistent with the heavy effective mass of electron pockets. By using the relationship between D_{e} and the effective mass: $D_{\mathrm{e}} = 6\frac{k_{\mathrm{F}} m_{\zeta,\eta}^*}{\pi^2\hbar^2}$ for a spherical FS, we obtain the rough estimate of $m_{\zeta,\eta}^*$ for the electron bands, corresponding to $m_{\zeta,\eta}^* = 0.4m_0$, where the factor six is the number of electron pockets in the BZ, and we used the carrier density of electrons $n_{\mathrm{e}} \sim 1.77 \times 10^{20}$ cm^{-3} obtained in Ref. S1 to estimate k_{F}. We note that the effective mass is heavier than those for the hole pockets but underestimated compared to the DFT calculations (Fig. S4), which stems from the fact that the electron pockets, ζ and η, are anisotropic in shape (Fig. 1(i)), and the effective cyclotron mass is also anisotropic with respect to the applied magnetic field (Fig. S4(b)).

C. de Haas-van Alphen oscillations

We also observe the de Haas-van Alphen oscillations in the magnetization torque (τ). Figure S6(a) shows the field dependence of τ at various temperatures, where the B is canted by $\theta = 14^\circ$ from the c axis. The FFT of the background subtracted component $\Delta\tau$ is shown in Fig. S6(b). As in the case of the SdH oscillation, α and β branches are hard to be resolved, while γ and δ branches can be fitted with two Gaussian functions. The temperature dependence of each oscillation amplitude (Fig. S6(c)) is analyzed by Eq. (S1), and the obtained physical

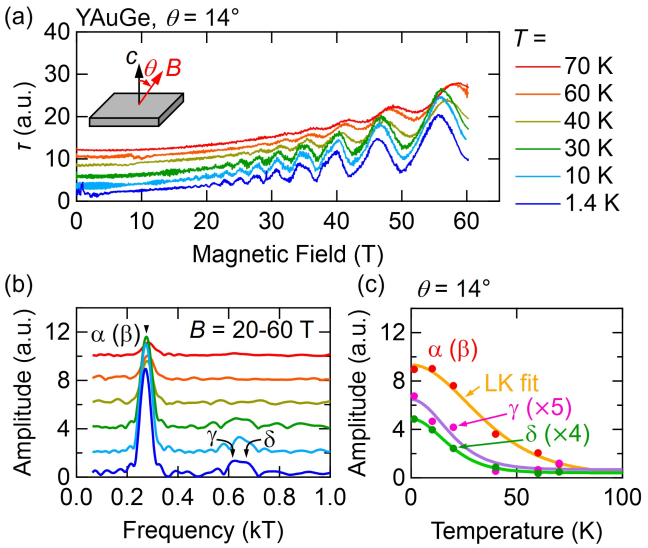

FIG. S6. (a) Field-dependence of magnetization torque (τ) at various temperatures with a magnetic field tilted from the c axis by $\theta = 14°$. Curves for $T > 1.4$ K are shifted for visibility. (b) FFT of dHvA oscillations of τ in the field range $B = 20-60$ T. (c) Temperature dependence of peak amplitude (closed circles) and fits with LK formula (solid lines) for each branch.

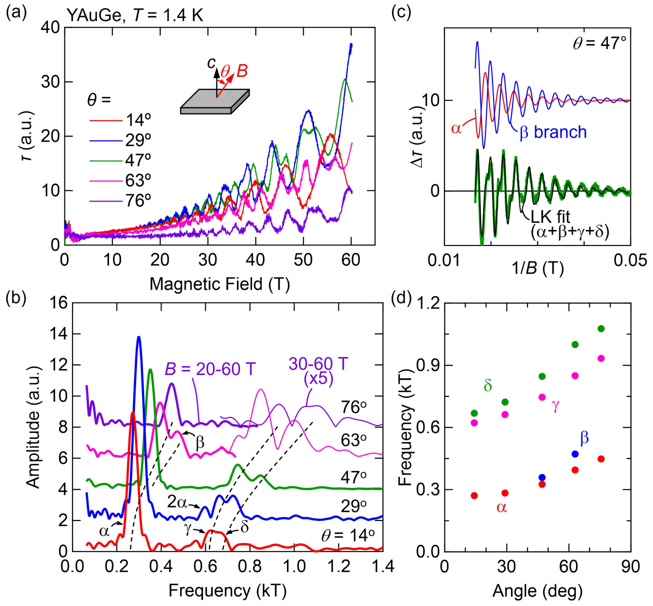

FIG. S7. (a) Field dependence of τ with various θ at $T = 1.4$ K. (b) FFT of dHvA oscillations in the field range $B = 20-60$ T (thick) and $B = 30-60$ T (thin). Dashed curve is guide to the eye. (c) Comparison between dHvA oscillations at $\theta = 47°$ and the LK fit. Thin black curve is the fitting result expressed by the summation of oscillation components for α, β, γ, and δ. Thin red (blue) curve is the oscillation component associated to α (β). (d) Angular (θ) dependence of the dHvA oscillation frequency for each branch (closed circles).

parameters such as the effective mass are summarized in Table S3.

The angular (θ) dependence of the de Haas-van Alphen oscillations is shown in Fig. S7(a). The FFT in Fig. S7(b) clearly resolve the branches γ and δ at higher angle. As for the α and β branches, we observe their splitting at $\theta = 63°$. The oscillation profile for $\theta = 47°$ (Fig. S7(c)) is nonmonotonic in $1/B$, suggesting a beating between α and β branches as observed in SdH oscillations (Fig. 3(a)). To further resolve the oscillations, we fit the raw data with the LK formula, where the oscillation part ($\tau_{\rm osc}$) of the magnetization torque is given below [S10].

$$\tau_{\rm osc} = \sum_{i=\alpha,\dots} \sum_{p=1,\dots} N_{i,p} B^{3/2} R_T^{i,p} R_{\rm D}^{i,p} \sin 2\pi (pF_i/B + \phi_{i,p}), \tag{S5}$$

The extracted oscillation patterns for the α and β branches are shown in Fig. S7(c). The summation of those well reproduces the amplitude modulation of the main oscillation in the raw data. The angular dependence of the oscillation frequenceis is plotted in Fig. S7(d), which is in good agreement with SdH oscillations. The FS parameters are summarized in Table S3.

D. SHUBNIKOV-DE HAAS OSCILLATION IN HOAUGE AND ANOMALOUS HALL EFFECT IN RAUGE

We have performed SdH oscillation measurement in HoAuGe by using the DC magnet in NHMFL, which can apply a high magnetic field up to 31 T. In magnetic materials with magnetization M, the quantum oscillations are known to be periodic to $1/B$ with $B = \mu_0 H_{\rm ext} - N_{\rm D} M + M$, where $\mu_0 H_{\rm ext}$ is the external magnetic field and $N_{\rm D}$ is the demagentization factor [S13]. In HoAuGe, the difference between $\mu_0 H_{\rm ext}$ and B is around 0.7 T when the saturation moment $10\mu_{\rm B}$ of a free Ho^{3+} ion is along the c axis. Due to the lack of knowledge on the magnetization curve above 7 T, an accurate B vs. $\mu_0 H_{\rm ext}$ curve is absent. The data is analyzed with $\mu_0 H_{\rm ext}$ instead of B. The oscillation frequencies are, thus, underestimated by $\sim 5\%$ in maximum.

Figures S8(a)-(c) summarize the temperature dependence of SdH in HoAuGe. Four oscillation frequencies are resolved by the FFT of $\Delta\rho_{xx}$ (Fig. S8(b)), which are close to the frequencies corresponding to the α, β, γ, and δ in YAuGe. The effective mass is estimated from the temperature dependence of the oscillation amplitude (Fig. S8(c)), which are comparable to those for YAuGe. These results confirm the rigid band approximation with respect to the substitution of R with Y. The Fermi surface parameters are summarized in Table S3.

Figures S8(d)-(e) show the SdH in HoAuGe at each angle of the magnetic field rotated from the c axis to the ab plane. Similarly with YAuGe, the quantum oscillations shift to higher frequencies as the magnetic field is tilted towards the ab plane. Figure 4(a) summarizes the

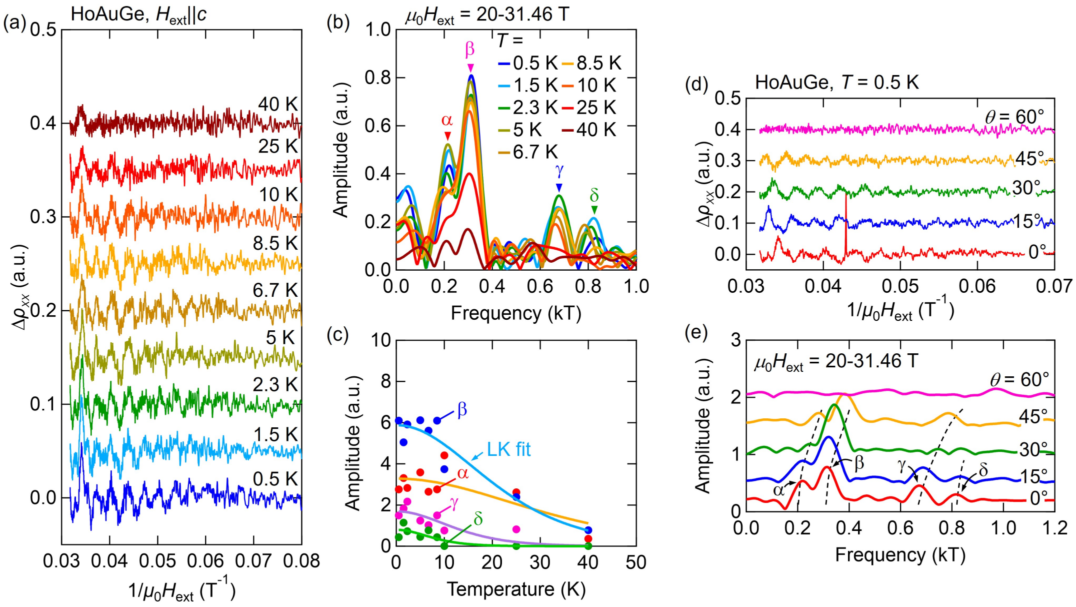

FIG. S8. (a) Inverse-field ($1/\mu_0 H_{\rm ext}$) dependence of the background-subtracted resistivity ($\Delta\rho_{xx}$) for HoAuGe measured at various temperatures with the B along the c axis. (b) FFT of SdH oscillations for $B \parallel c$ at various temperatures. Positions of the branches α, β, γ, and δ are assigned. (c) Temperature dependence of peak amplitude (closed circles) and fits with LK formula (solid lines) for each branch. (d) Inverse-field ($1/\mu_0 H_{\rm ext}$) dependence of the background-subtracted resistivity ($\Delta\rho_{xx}$) of HoAuge, measured at $T = 0.5$ K with the B tilted from the c axis to the ab plane by θ. (e) FFT of SdH oscillations at $T = 0.5$ K with various θ. Positions of the branches α, β, γ, and δ are assigned. Dashed curves are guide to eyes.

angular dependence of the oscillation frequencies.

We have performed the transport property measurement for RAuGe (R = Dy, Ho, Er, and Tm) by using the single crystals obtained in our previous study[S14], and have observed the anomalous magnetotransport responses. Figures S9(a)-(b) show the magnetic field dependence of the resistivity in the ab plane (ρ_{xx}) and the Hall resistivity (ρ_{yx}) for RAuGe (R = Dy, Ho, Er, and Tm) in $B \parallel c$ at $T = 1.8$ K. The demagnetization effect is corrected by using the magnetization data (M shown in Figs. S9(c), (f)), i.e., $B = \mu_0 H_{\rm ext} - N_{\rm D} M + M$. Compared to R = Dy and Ho as reported in Ref. S1, ErAuGe and TmAuGe show higher resistivity corresponding to the mobility of $\mu = 260$ cm^2/Vs and $\mu = 160$ cm^2/Vs, which are estimated by the Hall coefficient ($R_{\rm H}$) and the residual resistivity ρ_0 as $\mu = R_{\rm H}/\rho_0$. The negative magnetoresistance before the magnetization saturation in RAuGe (R = Dy, Ho, Tm) is mainly due to the magnetic phase transitions in the magnetic field or the field induced polarization. The origin of the positive magnetoresistance in ErAuGe has not been clarified yet. This might be related to the electronic structure change from the antiferromagnetic ground state to the field-induced polarized state. Further investigation of the magnetic structure is a subject of future study. The Hall coefficients are estimated from the slope of ρ_{yx} at the fields above the magnetization saturation, corresponding to the carrier density of 3.1×10^{20} cm^{-3} for ErAuGe and 3.5×10^{20} cm^{-3} for TmAuGe. The signature of the anomalous Hall effect in ErAuGe and TmAuGe can be seen in the nonmonotonic field-dependence of ρ_{yx} (Fig. S9(b)), while the sign of the AHE in TmAuGe is negative in contrast to those in other RAuGe (R = Dy, Ho, and Er).

We note that the anomalous Hall effect in DyAuGe and HoAuGe has already been reported in our previous study[S1]. In order to extract the AHC, we have applied the two-band model and introduced the field-dependence of the carrier mobility. For the magnetotransport properties in frustrated magnets, this procedure is valid for precise fitting parameters reproducing the field dependence of σ_{xx} and σ_{xy} simultaneously[S15]. The extracted $\sigma_{xy}^{\rm A}$ for DyAuGe and HoAuGe is shown in Fig. 4(f). We apply this analysis and extract the $\sigma_{xy}^{\rm A}$ in ErAuGe and TmAuGe. By using ρ_{xx} and ρ_{yx}, the conductivity and Hall conductivity are estimated as $\sigma_{xx} = \frac{\rho_{xx}}{\rho_{xx}^2+\rho_{yx}^2}$ and $\sigma_{xy} = \frac{\rho_{yx}}{\rho_{xx}^2+\rho_{yx}^2}$, respectively (Figs. S9(d)-(e) for ErAuGe, S9(g)-(h) for TmAuGe). Since the Hall mobility is small in ErAuGe and TmAuGe, we assume that the one-band model is sufficient to fit the field dependence of conductivities:

$$\sigma_{xx}^{\rm OB} = \frac{en\mu}{1+(\mu B)^2}, \tag{S6}$$

$$\sigma_{xy}^{\rm OB} = \frac{en\mu^2 B}{1+(\mu B)^2} + S_{\rm H} M, \tag{S7}$$

where n is the carrier density for the hole bands, and $S_{\rm H}$ is the constant for AHC ($\sigma_{xy}^{\rm A} = S_{\rm H} M$) We first fit the

field-dependence of σ_{xx} for $B > 4$ T by using Eq. (S6) with a constant μ. The fit result (thin green curve) is in good agreement with σ_{xx} for $B > 4$ T. In order to remove the discrepancy at low fields, which is due to the magnetoresistance below the magnetization saturation, we introduce the field dependence of μ as shown in Fig. S9(c), which reproduces the entire field-dependence of σ_{xx} (dashed gray curve in Fig. S9(d)). By using the parameters as the starting values, we fit the σ_{xx} and σ_{xy} with Eqs. (S6)-(S7) simultaneously. The fit result and the extracted AHC is shown in Fig. S9(e). We apply the same procedure to TmAuGe as shown in Figs. S9(f)-(h). The field dependence of σ_{xy}^{A} for each RAuGe is shown in Fig. 4(f).

[S1] T. Kurumaji, M. Gen, S. Kitou, and T.-h. Arima, "Metamagnetism and anomalous magnetotransport properties in rare-earth-based polar semimetals RAuGe (R = Dy, Ho, and Gd)," Phys. Rev. B **110**, 064409 (2024).

[S2] C. Bradley and A. Cracknell, *The mathematical theory of symmetry in solids: representation theory for point groups and space groups* (Oxford University Press, 2010).

[S3] J. Kruthoff, J. De Boer, Jasper Van W., C. L. Kane, and R.-J. Slager, "Topological classification of crystalline insulators through band structure combinatorics," Phys. Rev. X **7**, 041069 (2017).

[S4] J. Zhang, Y.-H. Chan, C.-K. Chiu, M. G. Vergniory, L. M. Schoop, and A. P. Schnyder, "Topological band crossings in hexagonal materials," Phys. Rev. Mater. **2**, 074201 (2018).

[S5] G. Chang, B. J. Wieder, F. Schindler, D. S. Sanchez, I. Belopolski, S.-M. Huang, B. Singh, D. Wu, T.-R. Chang, T. Neupert, *et al.*, "Topological quantum properties of chiral crystals," Nat. Mater. **17**, 978 (2018).

[S6] L. Wu, F. Tang, and X. Wan, "Symmetry-enforced band nodes in 230 space groups," Phys. Rev. B **104**, 045107 (2021).

[S7] Y.-M. Xie, X.-J. Gao, X. Y. Xu, C.-P. Zhang, J.-X. Hu, J. Z. Gao, and K. T. Law, "Kramers nodal line metals," Nat. Commun. **12**, 3064 (2021).

[S8] T. Finteis, M. Hengsberger, T. Straub, K. Fauth, R. Claessen, P. Auer, P. Steiner, S. Hüfner, P. Blaha, M. Vögt, *et al.*, "Occupied and unoccupied electronic band structure of WSe_2," Phys. Rev. B **55**, 10400 (1997).

[S9] G. Landolt, S. V. Eremeev, O. E. Tereshchenko, S. Muff, B. Slomski, K. A. Kokh, M. Kobayashi, T. Schmitt, V. N. Strocov, J. Osterwalder, *et al.*, "Bulk and surface Rashba splitting in single termination BiTeCl," New J. Phys. **15**, 085022 (2013).

[S10] D. Shoenberg, *Magnetic oscillations in metals* (Cambridge university press, 2009).

[S11] M. Bouvier, P. Lethuillier, and D. Schmitt, "Specific heat in some gadolinium compounds. I. Experimental," Phys. Rev. B **43**, 13137 (1991).

[S12] M. A. Avila and P. C. Canfield, "Anisotropic magnetization, specific heat and resistivity of $R\mathrm{Fe}_2\mathrm{Ge}_2$ single crystals," J. Magn. Magn. Mater. **270**, 51 (2004).

[S13] J. R. Anderson and A. V. Gold, "de Haas-van Alphen effect and internal field in iron," Phys. Rev. Lett. **10**, 227 (1963).

[S14] T. Kurumaji, M. Gen, S. Kitou, K. Ikeuchi, M. Nakamura, A. Ikeda, and T.-h. Arima, "Single crystal growths and magnetic properties of hexagonal polar semimetals RAuGe (R = Y, Gd-Tm, and Lu)," J. Alloys Compd. **947**, 169475 (2023).

[S15] T. Kurumaji, S. Fang, L. Ye, S. Kitou, and J. G. Checkelsky, "Metamagnetic multiband Hall effect in Ising antiferromagnet $ErGa_2$," PNAS **121**, e2318411121 (2024).

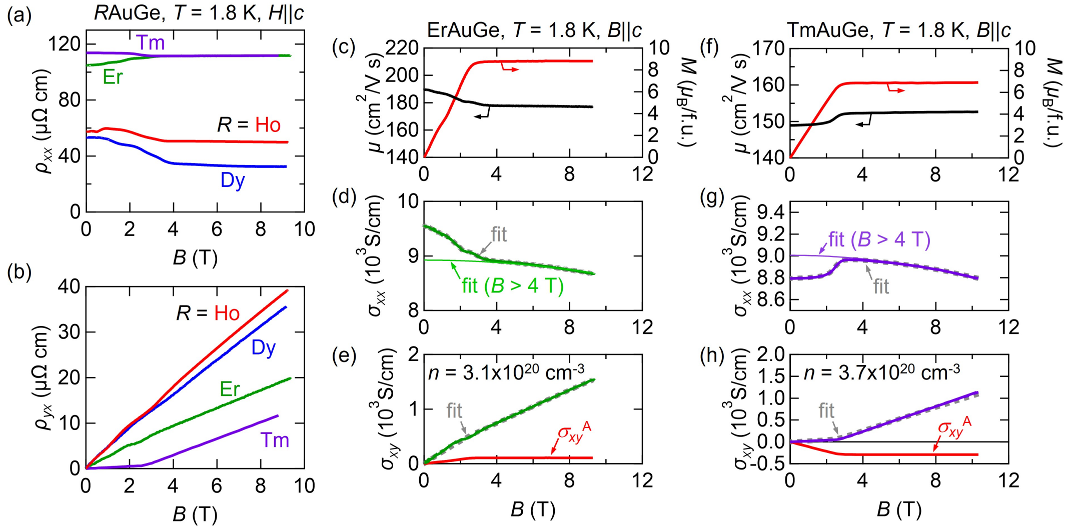

FIG. S9. (a)-(b) Magnetic field (B) dependence of (a) resistivity (ρ_{xx}) and (b) Hall resistivity (ρ_{yx}) in RAuGe (R = Dy, Ho, Er, and Tm) for $H \parallel c$ at $T = 1.8$ K. B is estimated by $B = \mu_0 H_{\rm ext} - N_{\rm D} M + M$, where $N_{\rm D}$ is the demagnetization factor. For DyAuGe and HoAuGe, the data is reproduced from Ref. S1 with permission. (c)-(e) B dependence of M, σ_{xx}, and σxy at $T = 1.8$ K in $B \parallel c$ for $R = Er$. Fitting results on the basis of Eqs. S6-S7 are also shown. (c) Carrier mobility μ and M, where M is saturated above $B = 3$ T and safely extrapolated from 7 T to the highest field; (d) [(e)] Thick green curve: observed σ_{xx} [σ_{xy}]; dashed gray curve: fit with Eq. XXX; thin green curve: fit for $B > 4$ T with a constant mobility; red curve: the AHC ($\sigma_{xy}^{\rm A} \propto M$). (f)-(h) Corresponding data for R = Tm.

TABLE S3. Fermi surface parameters of YAuGe and HoAuGe estimated by DFT, SdH, and dHvA measurements. $m_{\rm eff}$: effective mass; m_0: free electron mass; F: oscillation frequency; $k_{\rm F}$: Fermi wave number; $v_{\rm F}$: Fermi velocity; $\tau_{\rm q}$: quantum lifetime. θ is the angle between B (or $H_{\rm ext}$) and c axis. $k_{\rm F}$ ($v_{\rm F}$) is calculated by $2\pi eF/\hbar = \pi k_{\rm F}^2$ ($v_{\rm F} = \hbar k_{\rm F}/m_{\rm eff}$). LK denotes that the parameters are obtained by the fit of the raw curves with the LK formula (Eq. (1)). Otherwise stated, the parameters are obtained by the FFT. For $v_{\rm F}$ of β obtained by SdH oscillations in YAuGe, the $m_{\rm eff}$ of α is used.

Material	α	β	γ	δ
YAuGe (DFT, $B \parallel c$)				
$m_{\rm eff}/m_0$	0.128	0.140	0.246	0.248
F (T)	268	273	716	818
$k_{\rm F}$ (Å^{-1})	0.090	0.091	0.147	0.158
$v_{\rm F}$ (10^5m/s)	8.2	7.5	6.9	7.4
YAuGe (SdH, $B \parallel c$)				
$m_{\rm eff}/m_0$	0.10	-	0.21	0.25
F (T)	251	-	605	662
F (T) LK	247	286	605	654
$k_{\rm F}$ (Å^{-1}) LK	0.087	0.093	0.136	0.141
$v_{\rm F}$ (10^5m/s) LK	9.9	11	7.6	6.5
YAuGe (dHvA, $\theta = 14°$)				
$m_{\rm eff}/m_0$	0.13	-	0.23	0.24
F (T)	271	-	622	669
$k_{\rm F}$ (Å^{-1})	0.091	-	0.137	0.143
$v_{\rm F}$ (10^5m/s)	8.2	-	6.9	6.9
HoAuGe (SdH, $H_{\rm ext} \parallel c$)				
$m_{\rm eff}/m_0$	0.12	0.17	0.28	0.46
F (T)	217	314	672	816
$k_{\rm F}$ (Å^{-1})	0.081	0.098	0.143	0.157
$v_{\rm F}$ (10^5m/s)	8.0	9.7	5.8	4.0
